# Molecular Characterization and Phylogenetic Analysis of a Histone-Derived Antimicrobial Peptide Teleostin from the Marine Teleost Fishes, *Tachysurus jella* and *Cynoglossus semifasciatus*

**DOI:** 10.1155/2013/185807

**Published:** 2013-03-03

**Authors:** E. R. Chaithanya, Rosamma Philip, Naveen Sathyan, P. R. Anil Kumar

**Affiliations:** Department of Marine Biology, Microbiology and Biochemistry, School of Marine Sciences, Cochin University of Science and Technology, Fine Arts Avenue, Kochi 682016, Kerala, India

## Abstract

Antimicrobial peptides (AMPs) are host defense peptides that are well conserved throughout the course of evolution. Histones are classical DNA-binding proteins, rich in cationic amino acids, and recently appreciated as precursors for various histone-derived AMPs. The present study deals with identification of the potential antimicrobial peptide sequence of teleostin from the histone H2A of marine teleost fishes, *Cynoglossus semifasciatus* and *Tachysurus jella*. A 245 bp amplicon coding for 81 amino acids was obtained from the cDNA transcripts of these fishes. The first 52 amino acids from the N terminal of the peptide were identical to previously characterized histone-derived antimicrobial peptides. Molecular and physicochemical characterizations of the sequence were found to be in agreement with previously reported histone H2A-derived AMPs, suggesting the possible role of histone H2A in innate defense mechanism in fishes.

## 1. Introduction

Antimicrobial peptides (AMPs) have made their mark as an emerging class of natural antibiotics [1]. They represent molecules that have been retained by organisms throughout the course of their evolution as a part of defense mechanism against microbial enemies. Generally these gene-encoded ribosomally synthesized small peptides (<10 kDa) are cationic and very often amphiphilic moieties with selective broad spectrum antimicrobial activity [2]. The net positive charge enables these peptides to selectively attack negatively charged bacterial cell membranes and bring about destruction of the organism by pore formation or destabilization of membrane equilibrium or by penetration into the cell and thereby the impairment of cellular machinery [3]. This unique relatively irresistible mode of action and specific broad spectrum lytic activity to prokaryotes and virtual nontoxicity towards mammalian cells other than transformed cells make them attractive candidates as novel therapeutic agents [4, 5]. 

Aquatic organisms live in a microbe dominated environment where their protection from the austere external environment is enabled mainly by the mucosal barrier produced by the underlying epithelial cells [6, 7]. Fish mucus is known to contain a number of nonspecific humoral innate immune parameters including antimicrobial peptides [8–10]. Histone-derived antimicrobial peptides (HDAPs) and pleurocidins form two major groups of AMPs reported from fish mucus [9, 10]. Although the role of histone proteins as integral part of eukaryotic chromatin organization and as regulators of transcription is well known, their role in host immune responses is becoming more and more appreciated. The first report on the antimicrobial property of histones was from calf thymus [11]. There are two main types of histones: core histones (H2A, H2B, H3, and H4) and linker histone (H1), rich in either lysine or arginine residues [12]. Though HDAPs are reported from all five classes of histone proteins, H2A-derived AMPs lead the chart in terms of numbers. Parasin [8], hipposin [9], buforin I [13] and II [14], abhisin [15], himanturin [16], sunettin [17], and molluskin [18] represent histone H2A-derived AMPs, while onchorhyncin II [19] and SAM (Salmon antimicrobial protein) [20] are derivatives of histone H1. Antimicrobial properties of histone H2B [21], H3, and H4 are also well documented [12]. In the present study we report molecular characterization and phylogenetic analysis of a histone H2A-derived AMP, teleostin from Catfish *Tachysurus jella*, and Bengal Tongue Sole *Cynoglossus semifasciatus*.

## 2. Materials and Methods

### 2.1. Sample Collection

Live *T. jella* and *C. semifasciatus* were collected from Kalamukku harbour, Kochi, Kerala, India. Samples were transported to laboratory in live condition, and blood was collected from the lamellar artery near gill region using 5 mL syringes (RNase free) rinsed in precooled anticoagulant solution (RNase free 10% sodium citrate, pH 7). 

### 2.2. Total RNA Extraction and Reverse Transcription

Total RNA was extracted from the blood cells of the fishes with TRI Reagent in accordance with the manufacturer's instructions. Purity and quality of the RNA was analysed on 0.8% agarose gel, and the total purified RNA was quantified using spectrophotometer (A_260_ : A_280_). Only those RNAs having an absorbance ratio greater than 1.8 were used for the present work. The first strand cDNA was synthesised by reverse transcription in a 20 µL reaction mixture containing 5 µg total RNA, 1x RT buffer, 2 mM dNTPs, 2 mM oligo d(T_20_), 20 U of RNase inhibitor, and 100 U of M-MLV reverse transcriptase. The reaction was performed at 42°C for 1 h followed by an inactivation step at 85°C for 15 min. The efficiency of the reverse transcription reaction was verified using *β*-actin gene (primers used, F-5′-GATCATGTTCGAGACCTTCAACAC-3′, R-5′-CGATGGTGATGACCTGTCCGTC-3′) as control.

### 2.3. PCR Amplification

The PCR amplification of a hipposin-like gene from the cDNA of the fishes was performed in a 25 µL reaction volume containing 1x standard Taq buffer (10 mM Tris-HCl, 50 mM KCl, pH 8.3), 3.5 mM MgCl_2_, 200 mM dNTPs, 0.4 mM each primer, and 1 U Taq DNA polymerase using the forward primer (5′-ATGTCCGGRMGMGGSAARAC-3′) and reverse primer (5-GGGATGATGCGMGTCTTCTTGTT-3) [9]. The PCR condition involved an initial denaturation of 94°C for 2 minutes followed by 35 cycles of 94°C for 15 seconds, 60°C for 30 seconds, and 68°C for 30 seconds and a final extension at 68°C for 10 minutes. The amplicons were analysed by electrophoresis in 1.5% agarose gel in TBE buffer, stained with SYBR safe, and visualized under UV light. The PCR products were purified and sequenced at SciGenom, India.

### 2.4. Sequence Analysis and Molecular Characterisation

The sequences were analysed, trimmed, and assembled using GeneTool software. The cDNA-based gene sequences were translated using Expert Protein Analysis System (http://au.expasy.org/). Homology searches of nucleotide sequence and the deduced amino acid sequence were performed using BLASTn and BLASTp algorithm of the National Centre for Biotechnological Information (http://www.ncbi.nlm.nih.gov/blast). The physicochemical characterisations of the peptides were carried out using ProtParam tool (http://cn.expasy.org/cgi-bin/protparam) and the antimicrobial activity by Antimicrobial Peptide Data Base-Prediction (http://aps.unmc.edu/AP/main.php). The potential cleavage sites of various enzymes were predicted with Peptide Cutter tool of ExPASy. Previously reported histone H2A sequences of both vertebrate and invertebrate organisms were retrieved from GenBank and multialigned using ClustalW and GeneDoc computer programmes. Phylogenetic tree was constructed by Neighbor Joining method using the software MEGA 5.05. The three-dimensional structures of the peptides were predicted with the pdb data generated by SWISS-MODEL server using the software PyMOL. Helical properties of the peptide were delineated with Schiffer Edmundson wheel modelling using DNASTAR Lasergene 10.1 programme. 

## 3. Results

**Figure 1:**
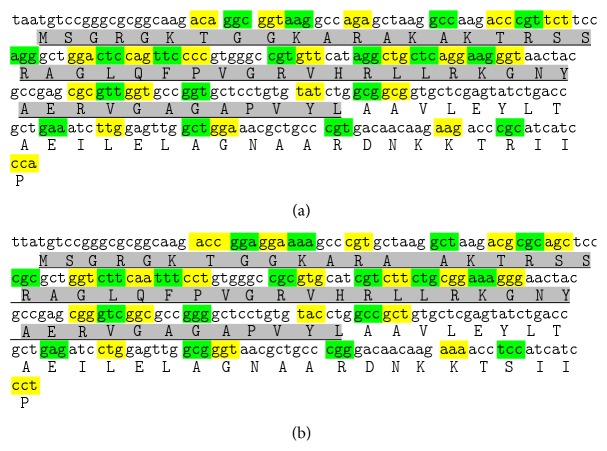
Nucleotide and deduced amino acid sequences of histone H2A from (a) *C. semifasciatus and* (b) *T. jella*. Differences in the two nucleotide sequences are highlighted in shades of yellow and green. The region corresponding to antimicrobial motif (teleostin) is indicated in grey.

**Figure 2:**
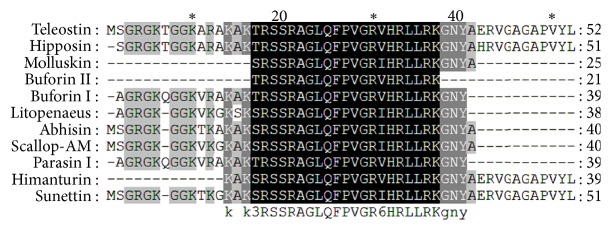
ClustalW multiple alignment of teleostin with previously reported histone H2A derived AMPs, from fishes and molluscs.

A 245 bp amplicon coding for 81 amino acids (aa) (Figure 1) was amplified from the blood cells of Bengal tongue Sole *C. semifasciatus* and Catfish *T. jella*. BLAST analysis confirmed the identity of sequences as histone H2A. The nucleotide and deduced amino acid sequence of the amplicon are shown in (Figure 1). Though differences in the nucleotide sequence of amplicons obtained from* C. semifasciatus* and *T. jella* were observed, the deduced amino acid sequences were found to be almost similar with a single exception at amino acid position number 78 where Arg in* C. semifasciatus *is replaced by Ser in *T. jella,* resulting from the degeneracy of the genetic code. The nucleotide and deduced amino acid sequence of the peptides have been submitted to GenBank (ID: HQ720152 and HQ720153, resp.). ClustalW multiple alignment (Figure 2) of the peptide sequences with previously reported HDAPs revealed that the first 51 amino acids from the N-terminus (excluding the initiator methionine) of the peptide were similar to the AMP hipposin reported from the skin mucus of Atlantic Halibut *Hippoglossus hippoglossus*. The only difference noted was His^41^ in hipposin, which is replaced by Glu^42^ in both the peptides reported from the marine bony fishes *C. semifasciatus* and *T. jella*. The Peptide Cutter tool of ExPASy predicted that the proteolytic enzymes chymotrypsin, pepsin, proteinase K, and thermolysin have cleaving sites at the position 52 of the peptide suggesting the possibility of formation of a 52 aa peptide similar to hipposin. Considering the exact similarity of amino acids between the peptides and high degree of conservation of the particular region among other teleost fishes this 52 aa peptide corresponding to hipposin was termed teleostin and hereafter will be referred by this term.

Peptide characterization using ProtParam tool revealed that the 52 aa Teleostin would have a predicted molecular weight of 5.527 kDa, a theoretical isoelectric point of 12.01, and a net positive charge of +12 contributed by 8 Arg and 5 Lys residues against a single Glu residue. The peptide was found to exhibit a total hydrophobic index of 32% contributed by amino acids Ala (13%), Val (7%), Leu (7%), Met (1%), and Phe (1%). The amino acids Gly and Arg have a higher percentage contribution to the total amino acids of the peptide, 17 and 15%, respectively. Protean programme of DNASTAR Lasergene 10 Core Suite predicts teleostin to have a concentration of 1.86 mg/mL for an absorbance of 1 OD at 280 nm, and 1 µg of the peptide would contain 180.52 pMol. Antimicrobial peptide database predicts teleostin to be an AMP with a protein-binding potential of 2.38 kcal/mol.

**Figure 3:**
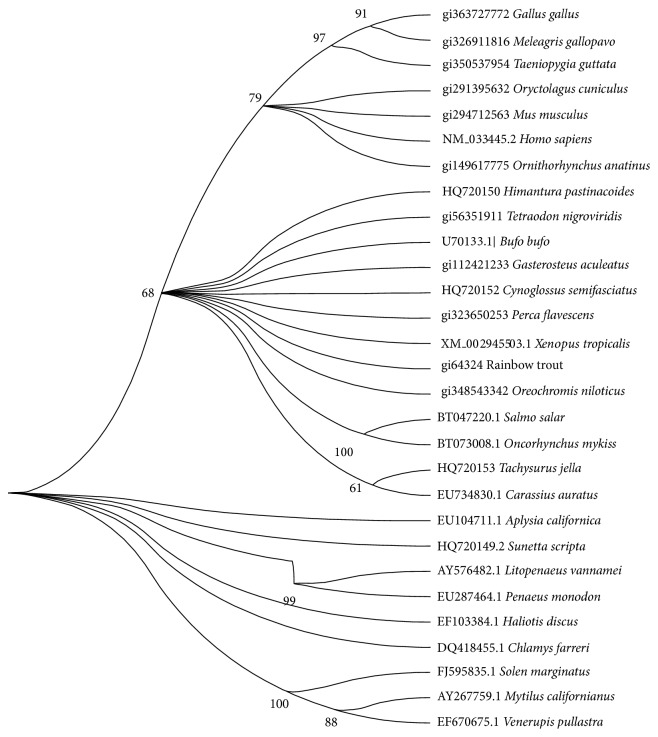
A bootstrapped neighbor-joining tree obtained using MEGA version 5.05 illustrating relationships between the nucleotide sequences of *Cynoglossus semifasciatus* and *Tachysurus jella* to the nucleotide sequences of previously reported histone H2A from various vertebrate and invertebrate organisms.

**Figure 4:**
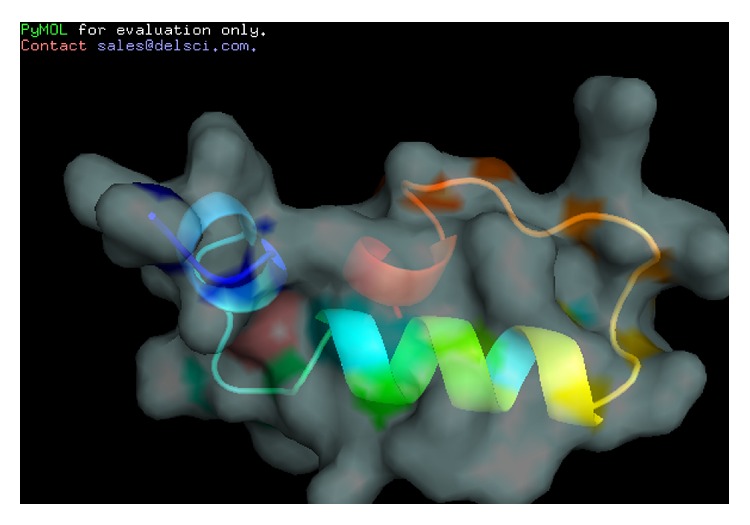
Predicted three-dimensional structure of teleostin, created using the software PyMol.

**Figure 5:**
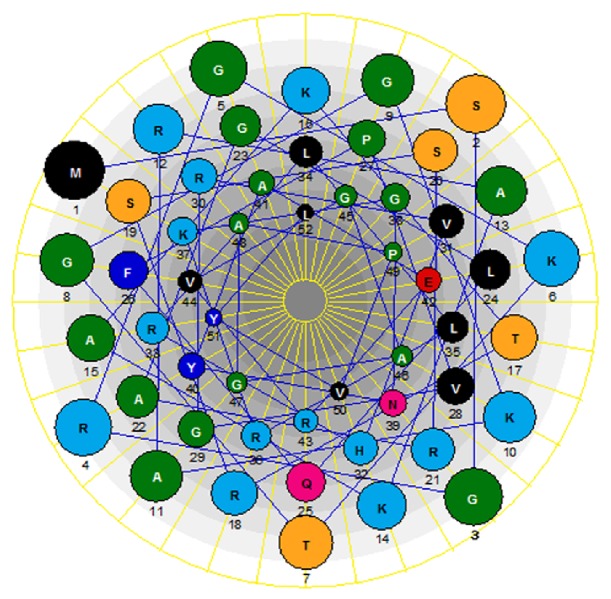
The Schiffer-Edmundson helical wheel diagram of teleostin created with DNAstar program demonstrating the possible amphipathic *α*-helical conformation. Helical wheel projects the arrangement of amino acids, and residue numbers are counted from the amino (N) terminus of teleostin.

Boot-strapped phylogenetic analysis based on the nucleotide sequence of histone H2A reported from invertebrates and vertebrates created a phylogenetic tree with three main clades, the first one representing invertebrates, the second one fishes and amphibians, and the third one birds and mammals (Figure 3). Teleostin aligned with the clade of fishes and amphibians, indicating its close resemblance with other teleost fishes. The software PyMOL predicted the structure of teleostin as an *α*-helix with two extended loops on either sides of the helix (Figure 4). The Schiffer-Edmundson helical wheel modelling further confirmed the helical structure of the peptide as the hydrophobic and hydrophilic residues were arranged on opposite sides of the wheel (Figure 5).

## 4. Discussion

Histone proteins are found to be rich in cationic amino acids, Arginine, or/and Lysine and hence have a net positive charge, which is the prime requisite for an AMP [1, 12]. Histone H2A is one among the core DNA-binding histones that is rich in Lysine residues. The N-terminus region of histone H2A is classically associated with DNA binding and is the region of nuclear signal localization [22]. Concurrently it is also reported to serve as a precursor for various AMPs [8–10, 13–20]. The paper deals with identification and characterization of a peptide (teleostin) containing an antimicrobial motif from the histone H2A of marine teleost fish *C. semifasciatus* and *T. jella*. 

Though fragmented histone proteins like hipposin have been detected from the skin mucus, the exact mechanism by which these peptides are formed is not yet known though there are reports indicating the role of proteolytic enzymes in formation of precursor-derived AMPs [8, 13]. We analyzed the histone H2A protein obtained from *C. semifasciatus* and *T. jella *for the cleavage sites of proteolytic enzymes using Peptide Cutter tool. The tool predicted the proteolytic enzymes chymotrypsin, pepsin, proteinase K, and thermolysin to have cleaving sites at position 52 from the N-terminus of the protein. The proteolytic activity of these enzymes on the protein in question would release a fragment similar to teleostin. Bacterial outer membrane proteases also assist in induced production of HDAPs, suggesting an infection-induced production of HDAPs [21]. Our hypothesis is in agreement with the study revealing the mechanism of production of HDAP parasin I from *Parasilurs asotus* which is mediated by matrix metalloprotease-activated cathepsin D upon the epidermal injury as a host defence mechanism against injury and imminent invasion of microbes [23]. 

The 52 aa teleostin has been characterized to be a cationic, amphipathic molecule that can configure to an *α*-helical conformation and represent the N terminal region of histone H2A protein. Except for the initiator Methionine and the replacement of the basic amino acid Histidine by an acidic amino acid Glutamine, teleostin is similar to the 51 aa HDAP hipposin that has been characterized from the skin mucus secretions of Atlantic halibut *Hippoglossus hippoglossus *[9]. Hipposin is an AMP devoid of any acidic amino acids and holds a net charge of +13. Teleostin was found to have a net charge of +12. The reduced cationicity of teleostin is mainly because of replacement of His in Hipposin by Glu in teleostin at position 42 which would reduce the cytotoxic activity of the peptide and make it more specific to acidic bacterial membranes [24]. Hipposin is among the most potent antimicrobial peptides discovered to date [9]. It has a broad spectrum activity against several Gram-positive and Gram-negative bacteria, and the activity could be detected down to a concentration of 1.6 µg/mL [9]. Since both teleostin and hipposin have similar composition of amino acids, the replacement of His by Glu in teleostin does not cause any conformational change to the helical structure and amphipathic content. Based on these similarities it could be concluded that teleostin will also have an activity comparable to the highly potent hipposin. 

The modes of action of AMPs will always be a matter of controversy, yet membrane disruption mediated osmotic imbalance, and subsequent bioenergetic collapse of the cell is the most convincing mode of action for AMPs [25]. Positively charged teleostin can easily interact with negatively charged target cell membranes and can bring about the disruption of membrane equilibrium by creating stable ion channels as in buforin I [26] or parasin I [27] or by direct disintegration and micellization of target cell membrane as in case of histone H2A isolated from trout mucus [28]. Helical property of the peptide has significant role in determining the antimicrobial activity [27]. Teleostin is an *α*-helical peptide, and hence it could interact with the hydrophobic phospholipid heads of the membrane and bring about the lysis of bacterial cells by cell membrane disruption. 

The enzyme cathepsin D produces parasin I in *P. asotus* [23]; similarly pepsin produces buforin I in *Bufo bufo gargarizans *and buforin II from buforin I by endoproteinase lys-C cleavage [26]. Specific proteolytic cleavage of 52 aa teleostin would give rise to three more peptides, a 19 aa parasin-like fragment, a 39 aa buforin-I-like fragment, and 21 aa residue buforin-II-like fragment corresponding to 2–20 aa, 2–40 aa, and 17–37 aa of the teleostin respectively. The 19 aa parasin-like and 39 residue buforin-I-like analogues of teleostin differ from that of parasin and buforin I in one and three amino acids, respectively, while the portion of teleostin analogous to buforin II is exactly the same. All the three fragments (19 mer, 39 mer, and 21 mer) derived from teleostin peptide sequence by predicted enzyme action were similar to the synthetic hipposin analogues of parasin, buforin I, and buforin II. Hipposin is the most potent histone H2A-derived AMP reported to date [29]. Teleostin being an analogue of hipposin would be expected to have an activity comparable to that of hipposin and therefore could be considered as a potent therapeutic agent and template for designing effective bioactive peptides with less cytotoxic effect and novel mechanism of action. 

The NJ phylogenetic tree based on nucleic acid sequences of histone H2A reported from wide range of organisms indicates a common ancestral origin of invertebrate and vertebrate histone proteins and their adaptive divergence to present positions. The tree branched into three main clades, and the histone H2A sequences of *C. semifasciatus* and *T. jella *aligned along with the clade formed by fishes and frogs. Significant overlap was observed in the histone H2A nucleotide sequences of “amphibians and fishes” and “birds and mammals” from which it could be inferred that histone proteins remain relatively unchanged throughout the course of evolution. 

## 5. Conclusion

Histone proteins and their derivative peptides play inevitable role in the defence of many organisms especially in fishes because they rely more on the rapid, nonspecific innate immune parameters than short-lived adaptive immune responses. The 52 residue *α*-helical cationic peptide teleostin identified from the marine bony fishes possess all characteristic features of classical AMPs. The 52 residue *α*-helical cationic peptide teleostin identified from the marine bony fishes possess all characteristic features of classical AMPs and has the potential to be developed into a novel therapeutic agent. Moreover they could be used as templates for the development of hybrid/stabilised AMPs.
